# How do genetically disabled adults view selective reproduction? Impairment, identity, and genetic screening

**DOI:** 10.1002/mgg3.463

**Published:** 2018-09-09

**Authors:** Felicity K Boardman, Rachel Hale

**Affiliations:** ^1^ Warwick Medical School Coventry UK

**Keywords:** attitudes, disability, genetic impairment, genetic screening, UK

## Abstract

**Background:**

Genomic medicine is rapidly evolving, particularly in the domain of reproduction. Population carrier screening for a range of disorders is becoming possible using whole genome/exome sequencing. However, very little is known about the views of genetically disabled adults toward selective reproduction.

**Methods:**

Forty‐three in‐depth qualitative interviews were carried out with adults living with different types of genetic condition, recruited through support groups and clinics. Interviews covered participants’ experiences of their condition and their views toward genetic intervention in reproduction. Thematic analysis of the data using NVivo 11 was undertaken, and participants were assigned categories as either supporting, not‐supporting, or having ambivalent views toward selective reproduction.

**Results:**

The majority of participants (65%) expressed either disapproval of, or held ambivalent views toward, selective reproduction. Key reasons for non‐support included regarding genetic impairment as part of personal identity and the prioritization of social and environmental barrier removal. Key reasons for support of selective reproduction included negative and externalizing attitudes toward genetic impairment and a belief in the importance of informed reproductive decision‐making.

**Conclusion:**

The degree to which participants identified with their impairment, more so than how they valued it, was significant in determining attitudes toward selective reproduction. Those who supported genetic screening viewed their impairment as separate to themselves, while participants who considered their impairment as integral to their identity were most likely to report ambivalent or negative attitudes. Policymakers and stakeholders considering the role of genetic carrier screening panels might usefully engage with adults affected by heritable disease as well as disability identity politics when considering the acceptability and social impact of genetic screening programs.

## 1 INTRODUCTION

Genomic medicine is a rapidly advancing field, and nowhere more markedly than within the domain of reproduction. Technologies such as whole genome sequencing (capable of screening individuals and fetuses for large panels of genetic disorders simultaneously) and noninvasive prenatal testing (NIPT, which renders the process of obtaining samples for such genomic screening both safer and more efficient than ever before) are increasingly being subsumed within mainstream health care (e.g., the 100,000 Genomes Project) bringing with them significant social and ethical challenges (Nuffield Council on Bioethics, 2018). Indeed, through the capacity to identify genetic carriers (i.e., people who may transmit a genetic disorder but who do not have one themselves) for increasingly large panels of genetic disorders (Plantinga et al, 2016), ideas about “responsible” reproductive decision‐making are, in tandem, rapidly evolving.

Currently, the birth of a child with an inherited genetic disorder is most commonly an unanticipated event. As such, reproductive decisions regarding subsequent pregnancies for the carrier parents are typically made in the context of their first‐hand knowledge about that particular condition (McClaren et al, 2010). Research has consistently demonstrated the significance and value of this “experiential knowledge” to families living with genetic disease in the formulation of their subsequent reproductive attitudes and decisions (Clarke, 2016; Dudding, Wilcken, Burgess, Hambly, & Turner, 2000). The introduction of preconception genetic screening, through the use of whole genome sequencing (WGS) or whole exome sequencing (WES), however, has the capacity to fundamentally alter this typical pathway through reprogenetic decision‐making (Plantinga et al, 2016). By alerting carrier parents to the possibility of their child having a genetic disease *before that child is even conceived*, such parents will, in the future, have the opportunity (not currently afforded to them within NHS care) of avoiding having a genetically impaired child altogether. This marks a significant shift. Reproductive decision‐making associated with rare and inheritable genetic disorders will no longer be the exclusive domain of people already familiar, or firmly embedded within, the realities of life with that condition. Rather, they will increasingly be made by members of the general public—the vast majority of whom will never have experienced, or even heard of the conditions before (Ioannou, Delatycki, Massie, Hodgson, & Lewis, 2015; McClaren, Delatycki, Collins, Metcalfe, & Aitken, 2008).

These changes, suggested by early population screens, are particularly significant for genetically disabled people. While disabled people and their families have long been described as the “best experts” on the condition(s) they live with (Petersen, 2006), the lack of open dialogue with such families and individuals around genetic interventions in reproduction is particularly striking, being highlighted as a significant concern in the Nuffield Council's ethical review of noninvasive prenatal testing (Nuffield Council on Bioethics, 2017). While this lack of inclusion might be justified on the grounds that already affected families are not the intended recipients of population‐level genetic screening programs, there is nevertheless the potential for significant impacts for them. Such impacts might include reductions in peer support (as less children come into the world with the same disorder), a decline in public funding into treatments and cures (due to the increased rarity of the condition), as well as the possibility of reduced social support for affected families as the public profile of the condition shifts from being viewed as an unavoidable occurrence, to a condition that is understood to be largely “preventable.” Indeed, the stigma of genetic disease and the “social policing” of families who have members affected by screened‐for conditions have been identified as having a significant and detrimental impact on the mental and physical well‐being families living with the condition (Clarke, 2016; Kellog, Slatterly, Hudgins, & Ormond, 2014). Moreover, the lived experiences of these families—a resource so pivotal to their subsequent reproductive decision‐making—is set to become increasingly scarce. As the number of births of affected children declines, so too does the rich “experiential knowledge” of these families. It is in this context of dwindling first‐hand experiential resources that potential parents will come to rely more heavily on the medical profession for information on the likely prognosis and daily impact of the conditions identified through screening programs. Whether or not the medical profession has adequate insight and resources to be able to offer this information on the lived experience of genetic diseases, however, is disputed both within and without the disability rights community (Farrelly et al, 2012; Williams, Alderson, & Farsides, 2002).

This paper takes the experiences of people living with various genetic disabilities as its point of departure in order to explore attitudes toward genetic intervention in reproduction. While the majority of the surrounding literature on reproductive decisions and attitudes within affected families have tended to focus on carrier parents (Al‐Jader, Goodchild, & Harper, 1990; Henneman, Kooij, Bouman, & Kate, 2002; Kelly, 2009; McClaren et al, 2010; Miller & Schwartz, 1992; Skinner, Sparkman, & Bailey, 2003; Watson, Williamson, & Chapple, 1991), a limited number of studies have emerged that consider the views of genetically disabled adults (both physically and cognitively impaired) toward prenatal diagnosis and selective reproduction. These studies have produced a complex picture, with some finding support (Chen & Schiffman, 2000; Conway, Allenby, & Pond, 1994; Janssens et al., 2016), reticence (Barter, Hastings, Williams, & Huws, 2016; Janssens et al, 2015; Kelly, 2009), and also ambivalence (Roadhouse et al, 2018; Stern et al, 2002; Taneja, Pandya, Foley, Nicely, & Arnos, 2004; Ward, Howarth, & Rodgers, 2002) toward genetic technologies. Despite these limited studies, there has nevertheless been a general lack of analytic interface between the way(s) in which people experience, and assign value to, their impairment, and how this relates to their views on genetic carrier screening for it.

By drawing on 43 in‐depth interviews with adults diagnosed with one of five highly contrasting genetic disorders, spinal muscular atrophy (*n* = 15), thalassemia (*n* = 8), hemophilia (*n* = 9), fragile X disorders (*n* = 2), or cystic fibrosis (*n* = 9), this paper presents a comparative analysis of the way in which people living with genetic impairment view the continuing evolution of genomic medicine in the domain of reproduction, and in particular, their views toward population‐level preconception and prenatal carrier screening for the condition they have themselves. In order to focus on the forms of screening that are most closely associated with selective reproduction, attitudes toward newborn genetic screening are not considered.

## 2 METHODS

### 2.1 Ethical compliance

Ethical approval for the study was granted through the Biomedical and Scientific Research Ethics Committee (REGO‐2017‐1910 21/02/2017) and the Health Research Authority (17/WM/0231 01/08/2017), respectively.

All participants in this study signed a consent form indicating that they had been fully informed about the nature of the interview, as well as the likely uses of their data. All names and identifiers were removed during transcription of the interviews. All names that appear in this paper are pseudonyms.

### 2.2 Methodology

The data drawn on within this paper are derived from two linked studies. The first (study 1) was a study of the views of families living with spinal muscular atrophy (SMA) toward different types of genetic screening, conducted between 2012 and 2015 (Boardman, Young, & Griffiths, 2017). Fifteen interviews with adults diagnosed with SMA have been included in this analysis. The second study (study 2), 2017–2020, involves four additional conditions, cystic fibrosis, hemophilia, thalassemia, and fragile X syndrome. Study 2 data were all collected between March 2017 and March 2018. These four additional genetic conditions were selected for inclusion in study 2 due to their comparatively high prevalence within the UK population as well as their contrasting (between conditions) and variable (within the diagnosis) presentations. Indeed, a broad range of impairment effects are associated with the five included conditions: both physical and cognitive/behavioral impairment effects, early‐ and late‐onset symptoms, treatable and nontreatable conditions, visible and hidden symptoms, fixed/static disability as well as emerging/degenerative impairment effects. This range of presentations was deemed pivotal for a comparative exploration of the relationship between experiences of the condition and attitudes toward selective reproduction using preconception or prenatal screening methods.

Interview participants were recruited primarily through condition‐specific support groups: SMA Support UK, The Haemophilia Society, The Fragile X Society, and the Thalassaemia Society. Calls for participants were placed in each of the groups’ electronic and postal newsletters, websites, and social media accounts (primarily Facebook and Twitter). Personal visits were also made by the researchers to the annual patient conferences of SMA Support UK and the Fragile X Society in order to distribute information about the study and answer questions about participation. Two interviews were conducted at both of these patient conferences.

Due to the preferences of the Cystic Fibrosis Trust, participants with cystic fibrosis were all recruited through a large adult Respiratory Medicine clinic in the north of England, supported by the Cystic Fibrosis Trust. Fifteen adults with cystic fibrosis were approached by a research nurse during routine clinic visits at the center and provided with a participant information leaflet about the study. Potential participants were then invited to contact the researcher to ask questions and/or arrange participation. This sampling technique leads to the successful recruitment of nine participants.

Interviews were conducted via three principle methods, face‐to‐face interviews (*n* = 6), telephone interviews (*n* = 19), and email interviews (*n* = 1). The choice of interview method was largely determined by the participant's preferences, abilities, and health status; however, due to the geographical dispersion of the participants throughout the United Kingdom, all participants living beyond a 150 mile radius of the University were offered a telephone, email, or Skype interview only. Face‐to‐face interviews were carried out at a variety of locations to suit participants. These included participants’ homes, coffee shops, a hotel, support group conferences, and a hospital. They lasted, on average, for approximately 60 min and were transcribed verbatim with names, place names, and any other identifiers removed.

The data were analyzed using NVivo 11 qualitative data analysis software. Open “broad brush” coding of the text was initially conducted to identify the core meta themes (e.g., “stories of diagnosis,” “day‐to‐day living,” and so on), before refinement of the codes was undertaken to develop more precise subthemes, supported by use of a hierarchical coding framework. Analysis continued to the point that “data saturation” had occurred; that is, no new themes were being added to the coding framework (Glaser, 1967).

In order to understand the relationship between experiences of genetic disease and attitudes toward genetic carrier screening, each participant was additionally categorized according to their degree of support for, or disapproval of, the introduction of genetic screening programs. The transformation of qualitative data into quantitative categories is acknowledged as a particularly useful mixed‐methods technique in order to undertake basic quantitative analysis (Sandelowski, Voils, & Knafl, 2009). The three categories used to group all 43 participants were as follows: “supports selective reproduction,” “nonsupport of selective reproduction,” or “ambivalence around selective reproduction.” In order to undertake the categorizations, a previously developed mixed‐methods technique of data summarizing was used (Boardman et al, 2011). As participants were asked directly about their support for screening within their interviews, most of the data were contained a particular point in the transcripts. The summarizing of these data resulted in 43 short summaries, each constituting approximately seven of eight lines of text. The data summaries were then categorized. Where categorization was not clear, the participant's fully coded transcript was returned to, and further explored, in order to identify supporting contextual data. This additional exploration occurred for nine participants in total.

The quotations presented in the paper were selected on the basis of them particularly eloquently or effectively illustrating the themes under discussion rather than the conditions per se. As such, no quotations from participants with fragile X syndrome and hemophilia are included; however, the perspectives of these participants were instrumental in developing the thematic framework of the analysis. Furthermore, the categories “nonsupport of selective reproduction” and “ambivalent toward selective reproduction” have been collapsed for the purposes of this analysis to allow a more focused analysis of the reasons for and against intervention in reproduction from the perspective of affected adults.

### 2.3 The conditions

In order to contextualize the qualitative data presented within this paper, it is firstly necessary to understand the broad implications of each of the conditions for those who live with them as well as the patterns of their inheritance. These are outlined below.

#### 2.3.1 Spinal muscular atrophy

Spinal muscular atrophy (SMA) is an autosomal recessive inherited neuromuscular condition affecting the lower motor neurons in the spinal cord, called the “anterior horn cells” (Sugarman et al, 2012). These cells are vital for relaying nerve messages between brain and muscle to enable movement. When a person has SMA, these cells deteriorate which means that nerve messages cannot be properly “relayed” between brain and muscle. The person's muscles then begin to waste or “atrophy” due to lack of stimulation and use. SMA has a range of clinical presentations and severities (Markowitz, Singh, & Darras, 2012). Type I is the most severe form and usually results in infantile death before the age of 18 months. Type II onsets early in childhood and results in significant disability, although life expectancy is not greatly curtailed. Type III and IV SMA usually onset in adult life and generally progress slowly so that a person is eventually unable to walk. In 2016, the first therapeutic for SMA, Nusinersen, was approved for use in children with type I (severe) SMA. This treatment may lessen, rather than completely cure, the symptoms of SMA.

#### 2.3.2 Hemophilia

Hemophilia is an inherited bleeding disorder usually caused by a genetic defect that leads to a deficiency of clotting factor proteins in the blood (Peyvandi, Garagiola, & Young, 2016). It is typically inherited in an X‐linked pattern, meaning it is most frequently passed on to men through the maternal line. The shortage of clotting factors means that a person with hemophilia bleeds for longer than usual, and this can be into joints. While hemophilia predominantly affects men, women may experience a related form of bleeding disorder known as von Willebrand disease. The severity of the bleeding disorder depends on the level of clotting factor an individual has (Den Uijl et al, 2011). Treatment typically involves blood transfusions and the regular transfusion of clotting factor (prophylaxis). Life expectancy is generally not affected.

#### 2.3.3 Thalassemia

Thalassemia refers to a group of inherited blood disorders that affect hemoglobin. People with the condition produce either too little or no hemoglobin. This can make them very anemic, experience delayed growth, osteoporosis, and reduced fertility (women). There are several forms of thalassemia, which can be divided into alpha and beta types. Beta thalassemia major is the most severe type. Other types include beta thalassemia intermedia, alpha thalassemia major, and hemoglobin H disease (Cousens, Gaff, Metcalfe, & Delatycki, 2010). People who are a "carrier" of thalassemia, also known as having the thalassemia trait (or thalassemia minor), typically do not have any serious health problems themselves, but are at risk of having children with the condition. Thalassemia disproportionately affects people of Mediterranean, South Asian, South‐East Asian and Middle Eastern origin (Ryan et al, 2010). Currently, all pregnant women in the United Kingdom are offered screening for thalassemia carrier status.

#### 2.3.4 Cystic fibrosis

Cystic Fibrosis (CF) is an autosomal recessive condition that primarily affects breathing and digestion. There are many different gene mutations that cause cystic fibrosis, so each person with the condition will have different symptoms depending on their individual genetic makeup (Castellani et al., 2010). Some people struggle with lung function and infections, while others need regular enzymes with every meal to help them digest their food. Many people with CF will develop CF‐related diabetes in adolescence or adulthood, and fertility is often affected in men. Many people with CF go on to need lung transplantation. Life expectancy for a person with CF today is approximately 47 years (UK Cystic Fibrosis Registry, 2013). A newborn screening program to identify both babies with CF (and some carriers of the condition) was introduced in the United Kingdom in 2007.

#### 2.3.5 Fragile X syndrome

Fragile X syndrome (FXS) is a genetic disorder inherited in an X‐linked dominant pattern (Hunter et al, 2014). While not life‐limiting, it is associated with a range of developmental difficulties, including learning disabilities and cognitive impairment. In addition, individuals with FXS may experience anxiety, hyperactive behavior, attention‐deficit disorder (ADD), features of autism spectrum disorders, seizures, fertility problems (female), and issues with social interaction. Usually, men are more severely affected than women.

While FXS the most common form of the condition, “fragile X” actually refers to a family of three associated genetic conditions, depending on whether a person has the full mutation (resulting in fragile X syndrome) or a premutation (Hantash et al, 2010). People with a premutation may experience: fragile X tremor‐associated ataxia (FXTAS) or fragile X‐associated primary ovarian insufficiency (FXPOI), or be symptomless. Approximately 1 in 151 women carry the FMR1 premutation as do approximately 1 in 168 men (National Fragile X Foundation, 2018).

## 3 RESULTS

### 3.1 Participants

Twenty participants were men (44%) and twenty‐five women (56%), and the majority of the sample (71%) were of reproductive age (defined as being aged between 15 and 49, in line with World Health Organization definitions) at the time of their interview (Tables 1, 2, 3, 4). A little under half of the participants were parents themselves (42%), although this varied considerably across the disease groups, with the highest number of parents falling within the hemophilia group (70%) and the lowest among those diagnosed with thalassemia (13%). Parental status was relatively similar across the remaining groups (cystic fibrosis 40%, fragile X disorders 50%, SMA [all types] 40%). The association of thalassemia with infertility in women (Skordis, Christou, Koliou, Pavlides, & Angastiniotis, 1998) and the overrepresentation of women within this group may, at least in part, account for the significantly lower rates of parenthood within this group. Similarly, only one male out of the five with cystic fibrosis was a parent, which may be explained by the condition's association with male infertility.

**Table 1:** Support for selective reproduction among adults with cystic fibrosis

Participant number	Participant ID	Condition	Age	Sex	Parental status	Approves selective reproduction	Disapproves selective reproduction	Conflicted selective reproduction
1	CF002	Cystic fibrosis	32	M			X	
2	CF003	Cystic fibrosis	32	F	Parent		X	
3	CF0004	Cystic fibrosis	32	F		X		
4	CF0005	Cystic fibrosis	58	M	Parent		X	
5	CF0006	Cystic fibrosis	38	F	Parent		X	
6	CF0009	Cystic fibrosis	39	F	Parent			X
7	CF0010	Cystic fibrosis	28	M			X	
8	CF0011	Cystic fibrosis	47	M		X		
9	CF0012	Cystic fibrosis	21	F		X		
10	CF0013	Cystic fibrosis	30	M				X

**Table 2:** Support for selective reproduction among adults with hemophilia

Participant number	Participant ID	Condition	Age	Sex	Parental status	Approves selective reproduction	Disapproves selective reproduction	Conflicted selective reproduction
1	H001	von Willebrand	66	F	Parent			
2	H007	von Willebrand	23	F			X	
3	H008	Von Willebrand	52	F	Parent			X
4	H0012	Hemophilia A (severe)	51	M		X		
5	H0013	Hemophilia A (severe)	46	M	Parent			X
6	H0015	Hemophilia A (moderate)	83	M	Parent		X	
7	H0017	Hemophilia A (moderate)	57	M	Parent			X
8	H0018	Hemophilia A (moderate)	21	M			X	
9	H0019	Hemophilia A	46	M	Parent			X
10	H0021	Hemophilia	72	M	Parent		X	

**Table 3:** Support for selective reproduction among adults with thalassemia

Participant number	Participant ID	Condition	Age	Sex	Parental status	Approves selective reproduction	Disapproves selective reproduction	Conflicted selective reproduction
1	TH001	Thalassemia	46	M	Parent	x		
2	TH002	Thalassemia	36	F		X		
3	TH004	Thalassemia	36	M		X		
4	TH006	Thalassemia	45	M		X		
5	TH007	Thalassemia	25	F		X		
6	TH008	Thalassemia	36	F		X		
7	TH0012	Thalassemia	44	F		X		
8	TH0013	Thalassemia	35	F		X		

**Table 4:** Support for selective reproduction among adults with spinal muscular atrophy and fragile X

Participant number	Participant ID	Condition	Age	Gender	Parental status	Approves selective reproduction	Disapproves selective reproduction	Conflicted selective reproduction
1	SMA001	SMA type II	30	F			X	
2	SMA005	SMA type II	44	F			X	
3	SMA009	SMA type II	25	M				X
4	SMA011	SMA type II	45	M				X
5	SMA014	SMA type II	37	F	Parent			X
6	SMA020	SMA type II	34	M				X
7	SMA023	SMA type II	60	F			X	
8	SMA029	SMA type II	31	F			X	
9	SMA007	SMA type III	47	F	Parent	X		
10	SMA008	SMA type III	55	M				X
11	SMA015	SMA type III	36	F	Parent	X		
12	SMA021	SMA type III	38	F	Parent			X
13	SMA025	SMA type III	40	F			X	
14	SMA004	SMA type IV	45	F	Parent	X		
15	SMA016	SMA type IV	67	M	Parent	X		
1	FX0013	Fragile X Syndrome	26	F				X
2	FX003	FXTAS	74	F	Parent	X		

Overall, the majority of participants identified as white British (78%); however, as expected due to the ethnic prevalence of thalassemia, all eight participants with thalassemia described themselves as being from minority ethnic backgrounds. Three identified as British Asians (two as Pakistani and one as Bangladeshi), two as Arabic (one Iranian and one Lebanese), and the remaining three participants all described themselves as white European (Italian and Greek Cypriot).

### 3.2 Support for selective reproduction

In total, 17 (38%) of participants in this study reported that they were in full support of a genetic carrier screening program being introduced for the condition they live with, whether this be a preconception genetic screening program (PCGS) or a prenatal screening program (PGS). Support for genetic screening was spread out across the dataset (cystic fibrosis three participants [30%], fragile X syndrome one participant [50%], hemophilia one participant [10%], thalassemia eight participants [100%], and SMA four participants [27%]). The most significant contrast was between people with hemophilia (where only one person supported selective reproduction) and thalassemia (where all eight participants reported that they fully support selective reproduction).

Firstly, the accounts of participants who supported selective reproduction will be presented, before moving on to those who rejected, or had ambivalent feelings toward it. The subheadings used reflect the key themes that emerged under the broader categories of “support” or “nonsupport” of selective reproduction.

#### 3.2.1 Support for selective reproduction: informed choice

For participants who supported selective reproduction, genetic screening was conceptualized as an important tool, facilitating informed and responsible parenting decisions. In a domain of life previously considered to be governed by “pot luck” or chance, genetic screening was viewed as a tool through which control and order could be exercised. Aliya (TH007) is a 25‐year‐old Iranian woman who was diagnosed with thalassemia major as a young child. Having come to live in the United Kingdom after her diagnosis, Aliya's condition has been managed primarily through regular blood transfusions and medications, enabling her to live independently and work full‐time. For Aliya, however, the possibility of choice, control, and informed decision‐making was critical, particularly for conditions like thalassemia where the burden of treatment remains high and a cure elusive:

I think in general, knowledge is power‐ the more you know, the more you are able to weigh up the risks and benefits of something..[…]… and if I were given a choice, I would always choose to have a child without thalassemia than one with thalassemia….the ultimate goal [of reproduction], as I see it, is to have a healthy child, and if it's possible and doable then I think it's the most responsible thing you can do as a parent, as a human being…[…]…a long time ago at the children's hospital [undergoing blood transfusion] and there was a mother in the daycare and one of her children was also having a blood transfusion for thalassemia and she was pregnant with her next child. And I distinctly remember her saying you know “what will be will be, it's in God's hands” and that stuck with me because I always thought “no, I think we have much more control now, and we are able to make conscious decisions as human beings.” I just thought that attitude was totally irresponsible.

For Aliya, the very availability of genetic technologies conferred particular responsibilities onto prospective parents and instilled a social expectation that they should exercise them in order to prevent the transmission of genetic disease at all costs. By minimizing the complex and multifactorial decisions associated with genetic screening and testing to a simple choice between a child *with* thalassemia or a child *without* it, Aliya highlighted her belief in the prioritization of the health of any future children as governing any reproductive decisions.

#### 3.2.2 Supporting selective reproduction: the physical impact of genetic disease

It is noteworthy that all participants who fell into the category of supporting genetic screening viewed the condition they live with as being associated with diminished health and (often) poor quality of life. Chiara (TH0012) is a 44‐year‐old woman who was diagnosed with thalassemia in Sardinia before moving to the United Kingdom at the age of six. For Chiara, “ignorance” and lack of experience with the condition being screened or tested for meant that quality of life is not appropriately factored into reproductive decisions by would‐be parents to the extent that she felt it should;

I think there is so much ignorance and that is why so many children continue to be born with thalassemia in this day and age, and it's just not acceptable. You know, I think the problem is that parents are selfish and thinking only of their desire to have a child and not their child itself [if they do not use screening and selective pregnancy termination].Because even if thalassemia is nowadays well controlled and we have an open prognosis….it still takes its toll….you have your freedom but your freedom is always on a leash….you will soon need another transfusion. So that's something that for me has stopped me having kids, I wouldn't risk to have a child with any type of disability that will be a burden on their life…because I know what it means, and if you don't have a direct experience of that then you cannot really fully understand.

The vivid contrast that Chiara constructs between her first‐hand insights into life with genetic disease (“I know what that means”), and the “ignorance” of parents who continue to have children with thalassemia irrespective of this suffering, Chiara was able to assert her perspective and experiences as an authoritative resource on thalassemia over the views of such parents and determine the boundaries of “acceptable” reproductive behaviors. For Chiara, only people with thalassemia themselves can fully appreciate what it means to live with it, and this notion of privileged knowledge and insight into their condition reoccurred across the disease groups, both among those who objected to, but also those who supported the use of carrier screening. Beth (CF003), a 32‐year‐old mother of two young children who has cystic fibrosis herself commented, “Yeah nobody, I don't care what the doctor says or how long he's studied for, nobody will ever be able to understand what this experience is like except another CF. We're the only ones who can make these [reproductive screening] decisions accurately really.”.

#### 3.2.3 Support for selective reproduction: the social impact of genetic disease

However, genetic disease is not experienced in a social vacuum, and participants within this group, mirroring the literature, also highlighted the range of social, cultural, and environmental factors that interfaced with disease severity to produce particular experiences of it (Miller & Schwartz, 1992). References to stigma, shame, and blame emerged spontaneously in participants’ accounts (despite them not being directly asked about within the interview) and were most prevalent among those who supported genetic screening, those from ethnic minority groups and those who had contracted bloodborne viruses in the treatment of their condition (typically hepatitis B/C or HIV). Participants described being avoided or misunderstood within their communities (Nathan, H0019, a 46‐year‐old man with hemophilia), having job offers withdrawn following disclosure of their health status (Sapphira, TH0013, a 35‐year‐old woman with thalassemia) or being ridiculed or disvalued for their condition (Jane, SMA023, a 60‐year‐old woman with type II SMA). Fariha (TH008) is a 36‐year‐old Bangladeshi woman who was diagnosed with thalassemia shortly after birth. For Fariha, the stigma surrounding her condition was as significant in creating obstacles in her life as the condition itself, particularly in the context of arranged marriages:

The problem is that…..there is a lot of stigma attached [to thalassaemia] within the South Asian community and really about people that had any sort of medical condition really…. I think because the condition isn't as widely known in the community, so they just think “oh she's got a problem, let's not have to deal with that issue.” So I'd need to sort of notify a potential partner and their family about it, they'd have to get tested and it just creates a lot of issues that they wouldn't have to deal with with a non‐thalassaemic person. So I wouldn't be an attractive partner for someone, I'd be black listed…so to speak. And that goes for my brother too when he wants to [get married] and everyone else related to me‐ we all go down in value.

Five of the eight participants with thalassemia described the combined practices of arranged marriages and consanguineous unions as starkly highlighting the cultural stigma surrounding genetic disease, a stigma that extends beyond the diagnosed adult to their wider family group, typically siblings. While many Middle Eastern countries (such as Iran, Saudi Arabia) practice compulsory premarital genetic screening for prevalent genetic conditions such as thalassemia, such practices were described as not “translating” into the Western context (Aliya), and instead, the responsibility for disclosing genetic disorders in the family (and particularly for nonvisible conditions) fell squarely on the affected family. For many participants, this disclosure often resulted in difficult negotiations.

Sapphira (TH0013) is a 35‐year‐old woman of Greek Cypriot background. She was diagnosed with thalassemia as a young child and described feeling very aware (from childhood) that the genetic status of any future partner was significant if she wanted children, something that she felt certain she wanted from a young age. As an adult, however, Sapphira was already engaged to her (now) husband, Stefan, before he was screened for thalassemia carrier status. When Stefan's test confirmed that he was, in fact, a carrier of thalassemia (meaning that each pregnancy they conceive would have a 50% chance of being affected), Sapphira described her mother urging her to break off the engagement. At the time of interview, Sapphira and Stefan were part way through their third and final (NHS funded) PGD cycle in an attempt to conceive a child without thalassemia:

I think everyone should have carrier screening before you get pregnant really so that you can be made aware beforehand…… before you even meet the person you want to have children with, really, and certainly within the first year of your relationship so that you can break it off. Because it's too difficult when you're so far down the road and you're so invested, which for us….it was much too late by then, so we went ahead and got married even though my mother wasn't happy. I just think it could save so much heartache and trouble if people know straight away and then they can back out [of a relationship] quickly.

For Sapphira who was dedicated to both “eliminating” thalassemia but who also had religious objections to selective pregnancy termination, the use of preconception carrier testing and PGD was the only route to parenthood that she believed upheld her genetic responsibilities, even in spite of the intense physical, emotional, and practical burdens this placed upon her, in terms of the disapproval of her family, the physical implications of PGD for her health and the strain on her marriage.

The participants whose accounts have been presented within this section largely viewed genetic disease as a burden upon the life of the affected individual, and their family as well as wider society. This was both in terms of quality of life but also the social implications of stigma and responsibility. However, not all participants viewed genetic disease this way. Indeed, the vast majority expressed ambivalent or negative views about the possibility of screening, accounts that will now be turned to.

### 3.3 Nonsupport or ambivalence toward selective reproduction

In total, 28 participants (65% of sample) reported concerns or objections about the introduction of genetic screening for the condition they live with. The reasons for these attitudes were complex and nuanced, drawing on a rich array of resources, primarily their lived experiences of genetic disease (Boardman, 2014a).

#### 3.3.1 Nonsupport for selective reproduction: valuing life affected by genetic disease

Indeed, the most common reason for screening rejection within this group related to a perceived mismatch between the way in which society views and values their genetic impairment in comparison with the way in which they actually experienced it in their lives.

Seth (CF002) is a 32‐year‐old man, diagnosed with cystic fibrosis (CF) at the age of two. Seth works part‐time as a doctor in a busy inner‐city hospital, a schedule which allows him time to physically recover from his shifts and also to manage his medication regimen. While Seth described CF as placing some limitations on his life, “I wouldn't say it reduces my quality of life, but I would say it affects my ability to take advantage of life,” he was very clear that he saw a contradiction between championing and affirming the lives of people with CF (including his own), “I think attitude is everything and we have to be positive about what people with CF can offer,” while also lending support to genetic screening, a practice which he acknowledged could have prevented his existence;

I can understand why somebody might want to find out whether their child was likely to have a genetic disease or an inherited malformation…such as Down's Syndrome [sic], but I struggle to…if it were me, to use that information to make a decision about whether or not to proceed with a pregnancy. But again I do think Down's syndrome [sic] in a kid would be much harder to deal with than CF….but it's just too close to home and too hypocritical for me to think it's ok to abort someone with one condition when I've also got a condition you can screen for and that is genetically inherited because that then opens the door to someone aborting me, or someone else with CF. …I just couldn't choose to abort a child based on the presence or absence of genetic disease because with that you're implying that one life is more worthy than another because of their genetics and that's an idea I can't get on board with….obviously.

Developed initially as a way to describe a particular response to prenatal testing and selective termination, the “expressivist objection”; that is, the idea that the practices of selective reproduction expresses disvalue of people with disabilities (Boardman, 2014a, 2014b; Parens & Asch, 2000) was frequently alluded to by people with different genetic conditions. For Seth, the practice of screening and the concomitant offer of pregnancy termination merged the diagnostic boundaries between screened‐for conditions. Down syndrome and cystic fibrosis became amalgamated in Seth's response as all “screened‐for conditions,” which meant that a termination for Down syndrome had implications for himself and other people with “detectable” disorders.

#### 3.3.2 Nonsupport of selective reproduction: identity politics of genetic disease

While it has been argued that the practices of selective reproduction reduce the genetically disabled fetus from being a “potential person” to a single genetic trait—that is, the disabling condition (Parens & Asch, 2000), participants in this study were keen to separate out the traits and characteristics they saw as comprising the personality of the person, from the condition that disables them. Henry (CF005) is a 58‐year‐old man who has lived with cystic fibrosis since infancy. While his condition rendered him infertile, Henry and his wife conceived a daughter through the use of donor gametes. For Henry, the use of a donor meant that he could be sure his daughter would not be a carrier of cystic fibrosis, “It's important for me that she don't have to worry about this stuff when it comes to her turn [to have children].” For while he would like to see a decline in the disease, he was ambivalent about genetic screening as being the means to achieve this.

In an ideal world, ok, no, you wouldn't want CF, so if they could get rid of CF through maybe gene therapy or drug therapy, then I think that would be great. But I just think people with CF have an awful lot to give…it's just a disability at the end of the day, it doesn't dictate how your whole life's going to be. Terminating CF babies would be just such a huge loss because who knows what them babies were going to bring? You know, eradicating CF is fantastic, but just not at the expense of abortions. Not at this price.

By recognizing that CF is merely one part of his life and not the whole, and just one part out of all the information there is to know about a fetus, Henry focused his critique of selective reproduction on its *reductionism* (i.e., the practice of valuing the fetus *only* as a CF fetus) rather than the nature (positive or negative) of that valuation per se. For Henry, CF remained a “cruel disease” a condition that “robbed” people of life and life opportunities, and consequently, he had few reservations about eradicating it. For him, selective reproduction is problematic not because it has the potential to reduce the incidence of CF, but because it does so by simultaneously eradicating those fetuses who would become people with CF.

#### 3.3.3 Nonsupport for selective reproduction: genetic disease as privileged state

Unlike Henry, however, some participants viewed their impairment in a far more positive light, as a uniquely valuable and integral part of their personhood. Sasha (SMA001) is a 30‐year‐old woman who was diagnosed with SMA type II at the age of 20 months. She never developed the ability to walk and has been using a powered wheelchair since the age of 4. Sasha works full‐time as a teaching assistant and lives independently with the support of personal assistants. Sasha described SMA as having given her a unique perspective on life that would otherwise have been inaccessible to her:

I just can't imagine my life without it [SMA], it's not only who I am, it's what I am. It's part of everything I do, from work, how I live what I dress like. And this is why screening for me is a no‐go for me personally, because I wouldn't have wanted to not come into the world just because of SMA and I wouldn't want someone making that decision for me. Because actually, it [SMA] gives me a unique perspective. It makes me relate to people on a level I just couldn't have done [without it]. I have insight, I suppose, into parts of human experience that no one in a million years…. I just think if someone said tomorrow “here's a cure,” you know, I would have to think very carefully about taking that, as odd as that sounds. And actually, I really don't know if I would.

It is noteworthy that all participants with SMA type II, a condition which is relatively static from birth, were more likely than those participants with degenerative forms of SMA (type IV or adult‐onset), or with a condition involving periods of ill health (such as CF) to express ambivalence or disapproval of selective reproduction. Shakespeare (2006), among others, Livneh and Antonak (2005), Boardman et al (2017), and Watson (2010), together with anecdotal accounts (Albers, 2018), have highlighted that personal identification with one's impairment is critical to the formulation of attitudes toward cure and elimination of disability. Those who remember their lives as an able‐bodied person or who experience periods of illness are likely to have very different views of their condition than people with fixed impairments (Boardman et al, 2017; Shakespeare, 2006), and these data confirm previous studies that this valuing of genetic impairment translates not only into attitudes toward cure, but also attitudes toward selective reproduction given that both are directed toward the goal of amelioration of genetic disease, albeit through very different approaches.

#### 3.3.4 Nonsupport of selective reproduction: the social construction of genetic disability

While difficulties associated with the stigma of genetic disease emerged as a key reason why selective reproduction was supported by participants in this study, it is noteworthy that it also appeared within justifications of the nonsupport of screening and selective pregnancy termination. While stigma was not specifically asked about within the interview schedule for this study, references to it nevertheless emerged spontaneously across the dataset both for those who supported selective reproduction, but also those who did not, highlighting the complexity of the relationship between genetic disease and stigma.

For participants who were uncomfortable with the practices of selective reproduction, stigma was no less prevalent than for those who supported it; however, for this group of participants, the existence of stigma underscored the social construction of genetic disease. Jonathan (SMA0011) is a 45‐year‐old man with type II SMA. He lives independently with the assistance of PAs and works as an accountant for his family's business. Jonathan was diagnosed with SMA shortly after birth following the diagnosis of his older brother, Lee (also with type II SMA) aged 18 months. For Jonathan, stigma was as much a part of his experience of genetic disease his impairment:

I honestly do think that 97% of the problems that I have in my day‐to‐day life are down to other people's attitudes than actually the SMA itself. I can manage the SMA, that's a piece of cake, but what we can't get away from the fact that we live in a society that views disabled people in a particularly negative way, excludes them and treats them as something to fear and avoid. So, no, I don't think abortion is the answer because you're making the disabled baby pay with its life the price for society's problem, actually…. So really if you think about it, the cure doesn't match the ailment does it?

By distinguishing between the biological domain of his impairment (the SMA) and its social consequence (his resulting disability), Jonathan, in line with social model of disability theorizing, suggested that social and political efforts to improve the lives of disabled people should invariably focus on latter rather than the former (de Wolfe, 2002). Selective termination was viewed by Jonathan as an entirely inappropriate response to what he saw as a primarily social issue. Indeed, a preference that funding invested in the implementation of genetic screening programs would be better spent on removing social, physical, and environmental barriers for disabled people was also mentioned by seven other participants within this group.

However, not all participants viewed their impairment through the prism of social model thinking and many instead highlighted the components of their impairment experience (typically pain, anxiety feeling unwell, uncontrolled bleeding) as being unamenable to social amelioration (de Wolfe, 2002). This is not to say that these participants did not place a high value on dismantling social barriers, but rather that they viewed particular aspects of their impairment experience as existing beyond their social construction of them. Furthermore, they viewed them as experiences for which an entirely inclusive and accessible society could not prevent, a caveat that some social model of disability theorists have more recently acknowledged (Shakespeare, 2006). While for many this could lead to negative, complex, or ambivalent attitudes toward the condition they live with (e.g., Henry), the data presented in this section highlight that, nevertheless, support and enthusiasm for selective reproduction remained a nonsequitur.

## 4 DISCUSSION

This paper is, to the best of our knowledge, the largest qualitative study of the views of people living with different types of genetic impairment toward the practice of selective reproduction. The literature has produced a somewhat contradictory picture of the views of this group of people, with some highlighting widespread support for genetic screening and testing (Chen & Schiffman, 2000) while others revealing more ambivalent views (Gollust, Thompson, Gooding, & Biesecker, 2003). This study highlights the complexity of the attitudes of genetically disabled adults and higher rates of ambivalence and negative attitudes than has previously been reported. As the capabilities of genomic technologies continue to evolve, the list of conditions they are capable of detecting in the preconceptual and prenatal period are expanding accordingly (Nuffield Council on Bioethics, 2018). It is within this context that the views of people living with “detectable” genetic diseases are set to become increasingly significant. Indeed, people living with genetic disorders are set to be dramatically impacted by the introduction of selective reproductive for their disease and its transformation into a “screened‐for” (and therefore potentially preventable) condition. Moreover, in the context of a strained NHS service, negative public attitudes toward conditions for which a person is deemed to have (wholly or in part) contributed to (such as obesity, type II diabetes, or smoking‐related diseases) highlight the significance of notions of personal accountability for one's health (Boardman et al, 2011). For individuals and their families living with genetic disease, the very availability of new methods of selective reproduction suggests new forms of “genetic responsibility” and accountability for decisions that previously did not exist (Hallowell, 2001).

As suggested by previous work with adults with genetic impairments, the data generated by this study highlight the significance of the way life with genetic disease is valued in determining attitudes toward selective termination (Gollust et al, 2003). As disabled people frequently rate the quality of their lives more highly than evaluations made by nondisabled people of them (Vimerö & Krause, 1998), it is perhaps somewhat unsurprising that the vast majority of participants within this sample expressed negative attitudes or ambivalence toward the genetic manipulation of reproduction. However, by including a range of disease groups, the data also highlight the plethora of factors that influence the degree to which a disabled person views their impairment positively. These include the social, environmental, cultural, and religious context, but also the degree to which a condition is internalized and regarded as a part of a person's identity (Watson, 2010).

The internalization of a disabled identity is a complex phenomenon, incorporating the nature, onset, and duration of lived experience of the condition (Boardman et al, 2017) as well as the way in which perceptions of the condition are reflected back to individuals through their encounters with the social world. Those participants who most ardently supported selective reproduction typically highlighted the negative nature of their impairment experiences (often describing periods of illness, pain, fatigue, and suffering) and the detrimental impact their condition had on their life, most commonly in terms of restricted life opportunities (most commonly in the domains of relationships, work, and housing) as well as lack of social, financial, and practical support. Intensive treatment regimes, particularly for those with CF and thalassemia, were also presented as prohibitive of a full and successful life in and of themselves as Chiara, a woman with thalassemia commented, “your freedom is always on a leash” (Chiara). It is notable that the stigma of genetic disease was also discussed more frequently by the 17 participants who supported selective reproduction than those who did not. This stigma was expressed in a variety of ways, as fear and avoidance of the affected person, but also as a reduction in status and prestige for those originating from communities within which arranged marriages and the integrity of bloodlines were highly valued. Indeed, it is noteworthy that all eight participants diagnosed with thalassemia (all from minority ethnic backgrounds) were in support of selective reproduction and three of these eight participants even argued that the prevention of future lives affected by thalassemia should be mandatory.

By separating out their sense of personal identity and self from their (negatively valued) genetic condition, these 17 participants could present selective reproduction as a straightforward decision between a disabled and an able‐bodied child. As one participant, Natasha (SMA004), a 45‐year‐old woman with type III SMA commented, “…it boils down to you having to choose your baby with SMA or without SMA, so I can't understand anyone choosing to let them keep the SMA.” This distinction is something of a misnomer given that—in the absence of a cure—a fetus and their genetic disorder cannot be, biologically speaking, separated out (thus, selecting against a genetically disabled fetus also means selecting against that *particular fetus* and replacing it with another, nondisabled fetus). However, by viewing their condition as something extrinsic to themselves, participants such as Natasha were able to navigate, and minimize, these more emotionally challenging parts of selective reproduction, including the expressive potential of reprogenetic decisions (Boardman, 2014a; Edwards, 2004).

For the majority of the sample, however, genetic disease was presented as being more deeply interwoven with personal identity—even for those who viewed their condition negatively (Henry). Selective reproduction was critiqued by these participants for its privileging of specific pieces of genetic information to determine the value of a whole individual, a tension that was easily recognizable for many participants given their broader experiences with disablism and stigma in everyday society.

This way of responding to genetic disease and selective reproduction allowed for more nuanced interpretations of participants’ lived experience. While for participants who supported selective reproduction, their condition could not be presented as anything other than a negative and invasive experience, for those whose condition was characterized as an integral part of their identity and sense of self, there was more room to interpret its impact in shifting and three‐dimensional ways. While Henry, like many of the participants who supported selective reproduction, described his lived experience of CF as often difficult and negative, by presenting it as an indelible part of his identity and daily life, his calculation of the costs of selective reproduction was markedly different from those of people who also negatively evaluated their condition, but identified with it in entirely different ways. .

As the empire of genomic medicine continues to expand and the voices of people with genetic disabilities become an increasingly scarce resource (Bricher, 1998), a sustained and in‐depth consideration of their views toward selective reproduction is now of critical importance (Nuffield Council on Bioethics, 2018; Scully, 2008). By exploring the perspectives of 45 people living with five very different genetic disorders, this study emphasizes that support for genetic intervention in reproduction is not universal among people living directly with the conditions that the technologies are targeted to prevent. Both support and nonsupport of selective reproduction hinged on the value each participant assigned to their quality of life and that which they assigned to others with the same condition. However, this study also highlights that this process of ascribing value was not a straightforward process whereby negative views of the condition *necessarily* implied support for selective reproduction. Rather, these judgments were thoroughly mediated by participants’ sense of their own self and personal identity and the position their condition occupied within this formulation. While the nature and type of impairment experiences participants reported had a significant and complex role to play within this calibration (Boardman et al, 2017), the data also highlight the significance of social and cultural factors in the way in which genetic impairments were internalized and valued, most notably the experiences of stigma and social support.

These findings suggest that even though notions of (medically defined) disease severity and quality of life are the concepts that continue to dominate policy discussions surrounding which conditions should (and which should not) be included on genetic screening panels (Korngiebel et al., 2016; Lazarin et al, 2014; Leo et al., 2016), that such stakeholder debates might more usefully attend to the complexity of disability identity politics and the social and cultural experiences of genetically disabled people (including that of stigma). If people with genetic impairments are to be meaningfully engaged in these debates, their relationship to their impairment is an important point of departure (Bricher, 1998). As this study highlights, it was not possible to “read off” participant attitudes toward selective reproduction by reference only to the nature and severity of their impairment. Genetic impairment is always experienced within a specific social, cultural, and environmental milieu which can be as significant in determining quality of life and reproductive attitudes for genetically disabled people as the condition itself.

### 4.1 Strengths and weaknesses

By including five different genetic conditions and utilizing a range of recruitment processes (including both clinics and support groups), this study offers an expansive view of the range of attitudes toward selective reproduction among people with genetic diseases. The data may be somewhat limited by the reliance on support group recruitment for participants with fragile X, hemophilia, thalassemia, and SMA; however, there were no marked differences in the responses between these groups and participants with cystic fibrosis, all of whom were recruited through an adult respiratory clinic. This suggests that the recruitment strategy did not significantly bias the findings.

Men with fragile X syndrome, who are generally considered to be most severely affected of all the fragile X disorders, are also missing from the sample. While attempts were made to recruit such participants for interview (typically after their parent had been interviewed), the degree of learning disability associated with fragile X syndrome was often described by parents as a barrier to their participation. While other research has successfully engaged adults with learning disabilities into research on selective reproduction (Barter et al, 2016; Ward et al, 2002), this perspective is missing from this study. Similarly, people with type I SMA (the most severe form) are absent from the sample due to the very poor prognosis associated with this form of the disorder. It is possible that the exclusion of these most severely affected individuals may have influenced the study findings. However, in spite of these limitations, the final sample nevertheless included a wide spectrum of disease presentations and severities, and, accordingly, also a broad range of perspectives on genetic screening.

## 5 FURTHER RESEARCH

Future research may usefully explore the way in which the development of gene therapies and genome editing influences the intersection of disability, impairment, and identity for people with genetic diseases. By obviating the perceived need for selective pregnancy termination, the possibility of gene therapy and/or genome editing is set to dramatically alter the landscape and purpose of selective reproduction. Research exploring what these developments mean for and to disabled people themselves will open up important lines of enquiry both within and beyond the selective reproduction debate.

## CONFLICTS OF INTEREST

The authors both declare they have no conflict of interests.
